# Individualized dosing parameters for tacrolimus in the presence of voriconazole: a real-world PopPK study

**DOI:** 10.3389/fphar.2024.1439232

**Published:** 2024-09-10

**Authors:** Yi-Chang Zhao, Zhi-Hua Sun, Jia-Kai Li, Huai-Yuan Liu, Bi-Kui Zhang, Xu-Biao Xie, Chun-Hua Fang, Indy Sandaradura, Feng-Hua Peng, Miao Yan

**Affiliations:** ^1^ Department of Pharmacy, The Second Xiangya Hospital, Central South University, Changsha, Hunan, China; ^2^ International Research Center for Precision Medicine, Transformative Technology and Software Services, Changsha, Hunan, China; ^3^ School of Basic Medicine and Clinical Pharmacy, China Pharmaceutical University, Nanjing, China; ^4^ Department of Urological Organ Transplantation, The Second Xiangya Hospital, Central South University, Changsha, Hunan, China; ^5^ School of Medicine, University of New South Wales, Sydney, NSW, Australia; ^6^ Centre for Infectious Diseases and Microbiology, Westmead Hospital, Sydney, NSW, Australia

**Keywords:** tacrolimus, population pharmacokinetics, voriconazole, renal transplantation, Monte Carlo simulations

## Abstract

**Objectives:**

Significant increase in tacrolimus exposure was observed during co-administration with voriconazole, and no population pharmacokinetic model exists for tacrolimus in renal transplant recipients receiving voriconazole. To achieve target tacrolimus concentrations, an optimal dosage regimen is required. This study aims to develop individualized dosing parameters through population pharmacokinetic analysis and simulate tacrolimus concentrations under different dosage regimens.

**Methods:**

We conducted a retrospective study of renal transplant recipients who were hospitalized at the Second Xiangya Hospital of Central South University between January 2016 and March 2021. Subsequently, pharmacokinetic analysis and Monte Carlo simulation were employed for further analysis.

**Results:**

Nineteen eligible patients receiving tacrolimus and voriconazole co-therapy were included in the study. We collected 167 blood samples and developed a one-compartment model with first-order absorption and elimination to describe the pharmacokinetic properties of tacrolimus. The final typical values for tacrolimus elimination rate constant (Ka), apparent volume of distribution (V/F), and apparent oral clearance (CL/F) were 8.39 h^−1^, 2690 L, and 42.87 L/h, respectively. Key covariates in the final model included voriconazole concentration and serum creatinine. Patients with higher voriconazole concentration had lower tacrolimus CL/F and V/F. In addition, higher serum creatinine levels were associated with lower tacrolimus CL/F.

**Conclusion:**

Our findings suggest that clinicians can predict tacrolimus concentration and estimate optimal tacrolimus dosage based on voriconazole concentration and serum creatinine. The effect of voriconazole concentration on tacrolimus concentration was more significant than serum creatinine. These findings may inform clinical decision-making in the management of tacrolimus and voriconazole therapy in solid organ transplant recipients.

## 1 Introduction

In the context of terminal renal failure, renal transplantation represents the most effective therapeutic intervention, offering the potential for significant improvements in both survival and quality of life (Turcotte, 1979; Vester et al., 1998). The management of renal transplantation recipients (RTRs) necessitates a complex immunosuppressive regimen, commonly involving calcineurin inhibitors such as tacrolimus or cyclosporine, alongside mycophenolate mofetil and corticosteroids (Vaden, 1997; Ciancio et al., 2004). Tacrolimus is a calcineurin inhibitor and a potent inhibitor of human T-cell proliferation (Naesens et al., 2009). In particular, is favored over cyclosporine due to its superior efficacy in promoting graft survival and its relatively more favorable side effect profile (Ciancio et al., 2004; Woodroffe et al., 2005; Bowman and Brennan, 2008). However, the clinical application of tacrolimus is complicated by its narrow therapeutic index and substantial intra- and inter-patient variability, which make precise dosing a persistent challenge for clinicians (Venkataramanan et al., 1995; Staatz and Tett, 2004; Davis et al., 2020; Degraeve et al., 2020). Achieving and maintaining therapeutic drug levels is critical, as deviations can lead to either graft rejection or drug toxicity, underscoring the necessity for meticulous dose optimization.

This challenge is further compounded in RTRs who are at an elevated risk for invasive fungal infections—a serious and often life-threatening complication in immunocompromised individuals (van Delden et al., 2020; Sharma et al., 2022). Azole agents, particularly voriconazole, are the cornerstone of therapy for these infections (Karthaus, 2010; Marr et al., 2015). However, the combination administration of tacrolimus and voriconazole is associated with significant pharmacokinetic interactions, most notably the inhibition of tacrolimus metabolism, which can lead to substantial fluctuations in tacrolimus blood levels (Kramer et al., 2011; Mori et al., 2012; Vanhove et al., 2017; Chen X. et al., 2021b). These fluctuations pose a critical risk to patient outcomes, necessitating careful management and dose adjustments to mitigate the potential for adverse reactions. Given the high prevalence of renal transplantation and the significant morbidity associated with improperly managed tacrolimus therapy, it is imperative to address the interaction between tacrolimus and voriconazole with precision. Although numerous pharmacogenetic and pharmacokinetic factors contribute to the variability in tacrolimus levels, the interaction with voriconazole is particularly significant and challenging to manage (Vanhove et al., 2017; Ota et al., 2019; Chen et al., 2022a). Current guidelines, including those outlined in the Vfend package insert, suggest reducing the initial dose of tacrolimus during voriconazole co-therapy. However, these these recommendations are often broad and lack specificity, resulting in inconsistent application across clinical settings. Furthermore, much of the existing literature has focused on the use of voriconazole in a general context rather than rigorously analyzing the specific impact of its dosage and concentration on tacrolimus pharmacokinetics (Vanhove et al., 2017; Chen et al., 2022a). This limitation underscores the need for a more nuanced and individualized approach to tacrolimus dosing, which remains a central concern for clinicians seeking to optimize therapeutic outcomes. Our previous study (Zhao et al., 2022), also provided critical insights into the interactions between these two drugs in RTRs, demonstrated that voriconazole significantly increases tacrolimus exposure and highlighted the importance of considering voriconazole concentration (C_VRC_) as a pivotal factor in the adjustment of tacrolimus dosing. However, while this study established a foundational understanding of the interaction, it also underscored the need for a more sophisticated modeling approach to guide clinical decision-making. Therefore, despite the widespread use of voriconazole in renal transplantation, there remains no consensus on the precise adjustments required for tacrolimus dosing during voriconazole co-therapy. By addressing this gap, our study seeks to provide clinicians with a practical and evidence-based tool to enhance patient outcomes through tailored tacrolimus management. Current study aims to develop a robust population pharmacokinetic (PopPK) model that incorporates C_VRC_ as a critical covariate, which can be used to simulate and optimize tacrolimus dosing in RTRs, offering a more precise and individualized approach to therapy.

## 2 Materials and methods

### 2.1 Study design and population

A non-intervention clinical study was conducted to investigate renal transplant recipients hospitalized at the Second Xiangya Hospital of Central South University between January 2016 and March 2021. The study received approval from the Ethics Committee of the hospital [(2020) Ethical Review [CR] No. (077)] and was registered on the Chinese Clinical Trial Registry (Registration number: ChiCTR2100048712). Throughout the study and data analysis, strict measures were implemented to maintain patient confidentiality.

Inclusion Criteria: 1) Patients who underwent renal transplantation for the first time; 2) At least 18 years old; 3)Hospitalized in the Renal Transplantation Department of the Second Xiangya Hospital; 4) Received voriconazole within 15 days post-operation; 5) Had at least three measurements of tacrolimus and voriconazole concentrations; 6) Received an oral triple immunosuppressive regimen of tacrolimus consisting of tacrolimus, mycophenolate mofetil, and glucocorticoid.

Exclusion Criteria: 1) Patients without precise records of tacrolimus dosage and duration; 2) Lacking cytochrome P450 (CYP3A5) and cytochrome P450 2C19 (CYP2C19) genotyping data; 3) Concurrently receiving cyclosporine, sirolimus, or other immunosuppressive agents; 4) On medications such as rifampicin, isoniazid, phenytoin sodium, or other strong CYP450 solid inducers or inhibitors; 5) Underwent renal replacement therapy within 72 h prior to concentration measurement.

### 2.2 Concentration and data collection

Tacrolimus concentrations in whole-blood samples were analyzed using a chemiluminescence microparticle immunoassay, specifically employing the ARCHITECT Tacrolimus Reagent Kit IL77-35, in accordance with the Prograf Assay Kit instruction il77-G08363R10-B1L77C (Laboratories, 2020). Voriconazole plasma concentrations were determined using the method previously described (Zhao et al., 2021). The National Health Commission Clinical Testing Center conducted annual laboratory quality evaluations to ensure the reliability of the results. Clinical data, including sex, weight, time post-transplant, age, albumin, hematocrit, creatinine (CREA), aspartate aminotransferase, C-reactive protein, total bilirubin, direct bilirubin, CYP3A5 genotype, and CYP2C19 genotype were collected. The tacrolimus administration schedule was determined by attending physicians based on clinical guidelines and their professional experience. We ensured that there was no interference with the established oral administration schedule of tacrolimus. Blood samples were obtained without intervention, with the majority collected within a 30-min window prior to tacrolimus administration.

### 2.3 Pharmacokinetic analysis and Monte Carlo simulation

Prior to developing the PPK model, we conducted an exploratory analysis of the data characteristics using the QQ plot, histogram, and frequency distribution diagram provided by the software. Baseline characteristics were summarized as mean (standard deviation: SD) or median (first quartile, third quartile), depending on their distribution, with categorical with categorical variables expressed as number (%). Subsequently, we employed Phoenix NLME pharmacokinetic software (version 8.1, Pharsight, a Certara Company, USA) to construct the PPK model for tacrolimus blood concentration-time data in RTRs receiving concomitant voriconazole. We assessed the model fit by calculating the Bayesian information criterion (BIC) and Akaike information criterion (AIC) to identify the most appropriate structural model (Vrieze, 2012; Liu et al., 2023). A shrinkage value below 20% was deemed acceptable (Xu et al., 2012). Covariate models were evaluated using a stepwise approach, starting with forward inclusion (*p* ≤ 0.01, ΔOFV >6.635) followed by backward elimination with more stringent criteria (*p* < 0.001, ΔOFV >10.828), ensuring consistency across the model. Model evaluation involved goodness-fit plots, bootstrap resampling, and visual predictive check (VPC). After finalizing the PPK model, we identified key covariates and used the Monte Carlo simulation method to predict tacrolimus trough concentration on the third day. In our study, the optimal dosage regimen was determined to achieve a target tacrolimus trough concentration within the therapeutic range of 5–10 ng/mL, with a probability of target attainment (PTA) of 70% or higher (Chen et al., 2022b).

## 3 Results

### 3.1 Study population and effect of voriconazole on tacrolimus concentration

**FIGURE 1:**
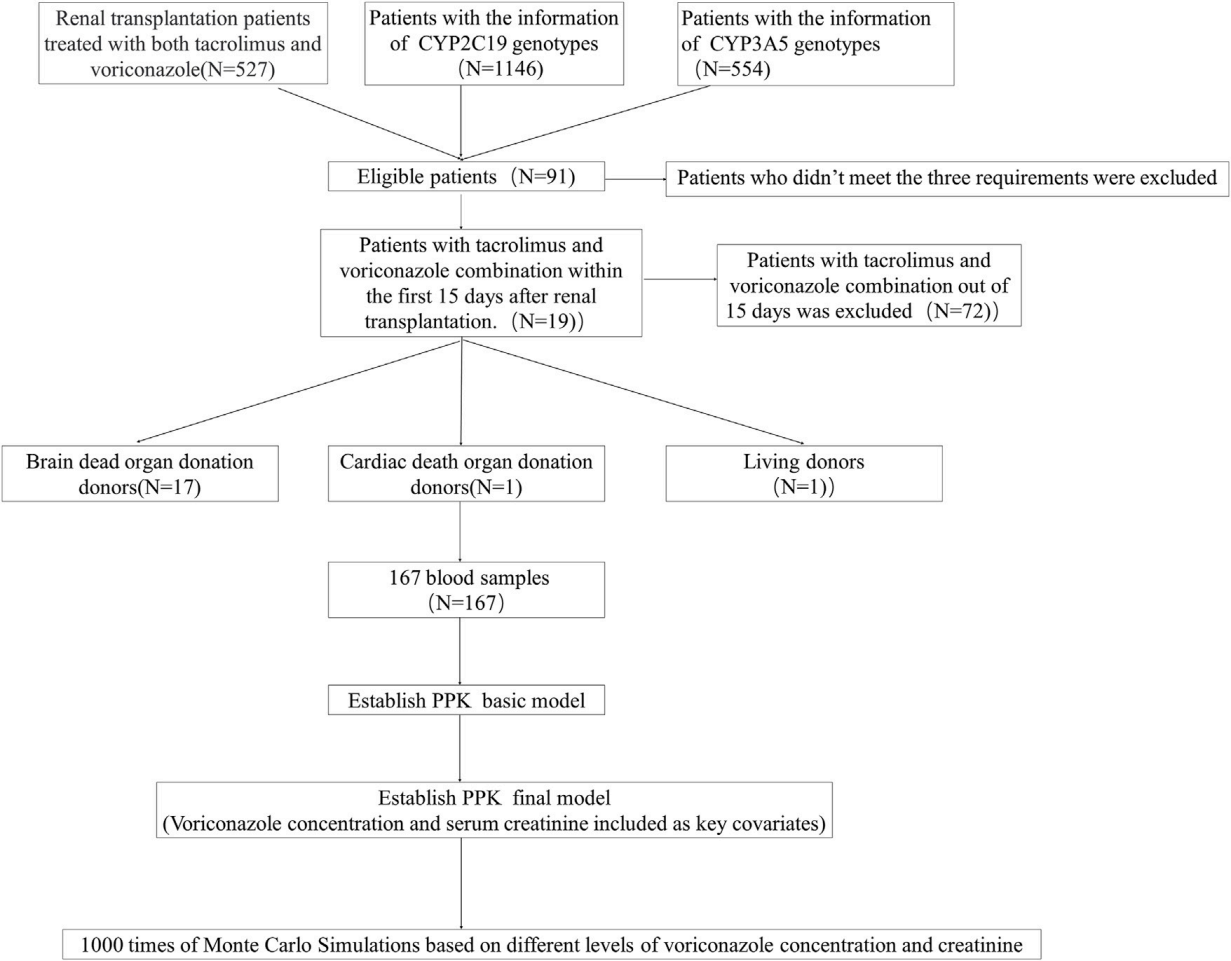
Flow chart of enrolled patients.

The study ultimately comprised 19 patients in the model-building group. The patients screening workflow is illustrated in the flow chart (Figure 1). Of these patients, 15 (78.9%) were male RTRs, with a median age of 44 years old and a median weight of 63 kg. Among them, 17 (89.5%) were donors from brain-dead organ donation (DBD), 1 (5.3%) was a donor from cardiac death organ donation (DCD), and 1 (5.3%) was a living donor. Only patients with the CYP3A5 genotypes *1/*3 (10 cases) and *3/*3 (9 cases) were included. In total, 167 blood samples were collected, with an average of 8–9 samples per patient. Detailed demographic data are presented in Table 1.

**TABLE 1:** Patient demographic data (N = 19).

Characteristic^a^	Level	Overall
Sex, n (%)	Male	15 (78.9)
	Female	4 (21.1)
Renal source, n (%)	DBD	17 (89.5)
	DCD	1 (5.3)
	Living	1 (5.3)
CYP3A5 genotypes, n (%)	^a^1/^a^3	10 (52.6)
	^a^3/^a^3	9 (47.4)
CYP2C19 genotypes, n (%)	^a^1/^a^1	6 (31.6)
	^a^1/^a^1	1 (5.3)
	^a^1/^a^2	9 (47.4)
Age (year)		44.00 [37.50, 52.50]
Weight (kg)		63.00 [51.00, 72.00]
Dose of tacrolimus (mg)		3.00 [1.50, 3.50]
Tacrolimus concentration (ng·mL^-1^)		7.90 [5.55, 10.78]
Time after operation (day)		8.00 [4.00, 11.00]
Voriconazole concentration (μg·mL^-1^)		0.00 [0.00, 0.50]
White blood cell count (10^9^/L)		8.54 [6.61, 10.61]
Red blood cell count (10^12^/L)		2.91 [2.64, 3.55]
Percentage of lymphocytes (%)		6.80 [4.30, 10.90]
Neutrophilic granulocyte percentage (%)		87.20 [80.50, 91.70]
Hematokrit (%)		26.20 [23.60, 31.40]
Hemoglobin (g/L)		88.00 [78.00, 103.00]
Platelet count (10^9^/L)		182.00 [148.00, 222.25]
Alanine transaminase (U/L)		10.50 [7.90, 18.80]
Aspartate aminotransferase (U/L)		12.10 [9.70, 17.63]
Total bilirubin (μmol/L)		7.20 [5.60, 9.10]
Direct bilirubin (μmol/L)		2.80 [2.10, 3.70]
Serum total bile acid (μmol/L)		3.30 [2.00, 6.25]
Albumin (g/L)		34.00 [31.70, 36.40]
Blood Urea Nitrogen (mmol/L)		25.83 [18.82, 38.36]
Serum creatinine (μmol/L)		237.00 [162.90, 648.00]

^a^ Measurement data are presented as median (interquartile range) and categorical data were expressed as frequencies; CYP, cytochrome P450.

### 3.2 Establishment of the PPK model

#### 3.2.1 Base model

In the structural model development process, both one-compartment and two-compartment models were evaluated for their Log-Likelihood, AIC, OFV, and shrinkage parameters. The results, summarized in Table 2, indicate that the performance of the one-compartment model was comparable to that of the two-compartment model. Additionally, the additive residual model yielded results similar to those of the proportional residual model.

**TABLE 2:** Comparison of base models.

Model description	LogLik	OFV^a^	AIC	BIC	Shrinkage (%)
1^a^ _Addictive	−495.11	990.2152	1,004.215	1,026.04	7.71
1^a^ _Multiplicative	−501.15	1,002.30	1,016.30	1,038.13	7.79
1^a^ _Add_Multiplicative	−492.85	985.70	1,001.70	1,026.65	6.59
Fixed Ka Model	−493.78	987.55	999.55	1,018.26	6.22
2^b^ _Addictive	−491.83	983.66	1,005.66	1,039.96	7.86
2^b^ _Multiplicative	−501.15	1,002.31	1,024.31	1,058.60	7.80
2^b^ _Add_Multiplicative	−489.17	978.33	1,002.33	1,039.75	4.18

^a^ First-order compartment model.

^b^ Two compartment model; OFV: objective function value; AIC: Akaike Information Criterion; BIC: bayesian information criterion.

However, given its increased complexity and additional parameters, the two-compartment model was considered less suitable. Consequently, a one-compartment model with first-order absorption and elimination, along with an additive residual model, was selected to describe the pharmacokinetic characteristics of tacrolimus. The base PPK model includes parameters for the elimination rate constant (Ka), apparent volume of distribution (V/F), and apparent oral clearance (CL/F), with Ka fixed during the analysis. The results of these parameters mentioned above are illustrated in Table 3. The values for Ka, V/F, and CL/F in the base model were 8.39 h^−1^, 5,291/L, and 32.14 L/h, respectively, with coefficients of variation (CV%) of 13.31%, 22.91%, and 18.05%. Following the removal of diagonal elements, the shrinkage value of CL/F and V/F were reduced by 22.5% and 3.87% respectively.

**TABLE 3:** Parametric results of the basic and final model^a^.

Parameter	Estimate	Units	Stderr	CV%	2.5% CI	97.5%CI
Base model						
Ka (Fixed)	8.39	1/h	0.00	0.00	8.39	8.39
V/F	5,291	L	0.91	17.18	3.50	7.09
CL/F	32.14	L/h	0.01	17.15	0.02	0.04
Final model						
Ka	8.39	1/h	0.00	0.00	8.39	8.39
V/F	2,690	L	0.33	12.35	2.03	3.35
CL/F	42.87	L/h	0.00	9.27	0.04	0.05
Θ_VRC-V_	−0.20		0.04	−18.52	−0.28	−0.13
Θ_VRC-CL_	−0.28		0.03	−9.41	−0.33	−0.22
Θ_CREA-V_	−0.40		0.11	−26.94	−0.61	−0.19

^a^ CV: variable coefficient; CI: confidence interval; V/F, apparent volume of distribution after oral administration; CL/F, apparent oral clearance; Θ_VRC-V_: exponent for C_VRC_, as a covariate for V:_ΘVRC-CL_: exponent for CVRC, as a covariate for CL; Θ_CREA-V_: exponent for CREA, as a covariate for V.

#### 3.2.2 Final model

**FIGURE 2:**
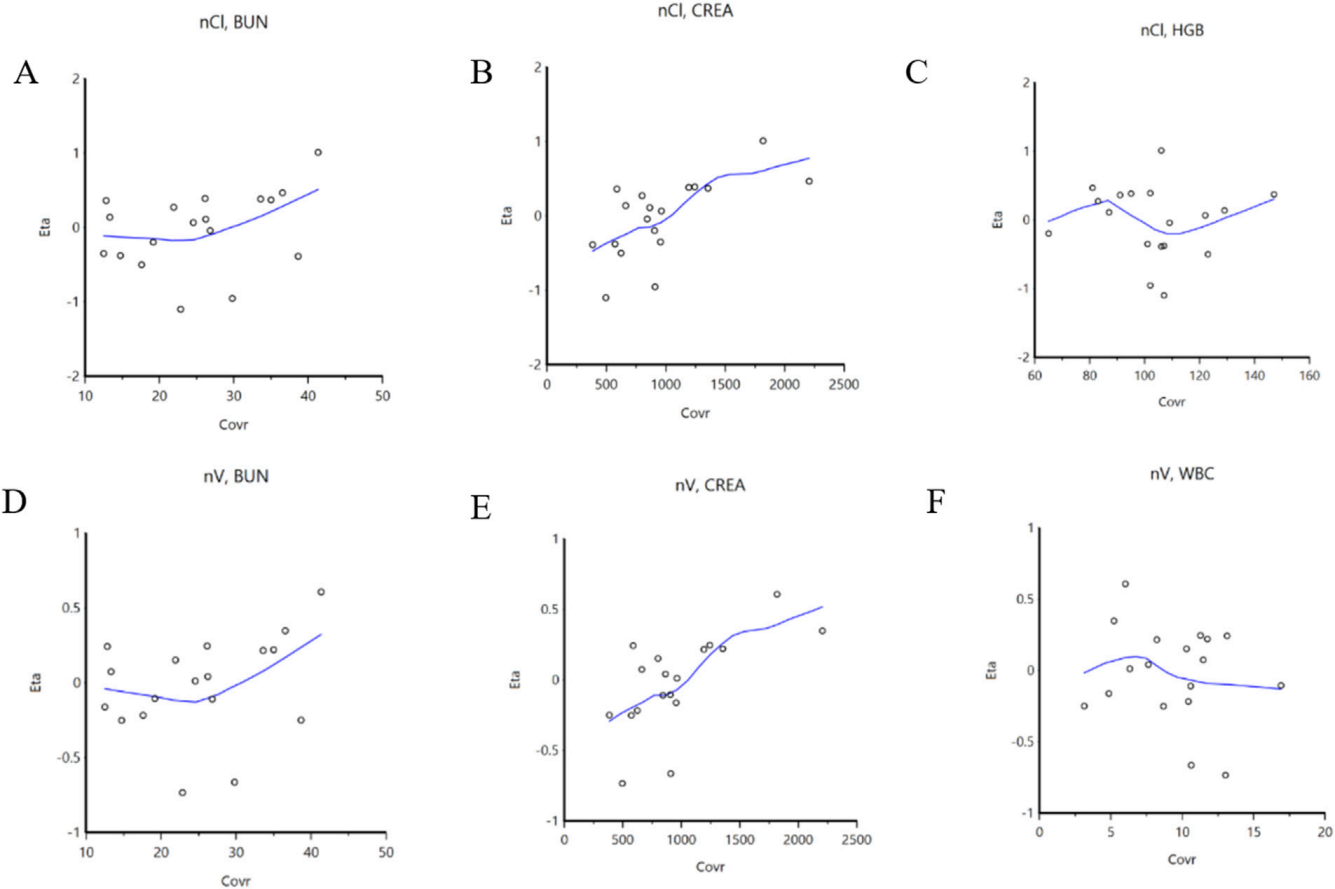
Correlation between covariables and parameters. **(A)**: BUN is correlated with CL/F; **(B)**: CREA is correlated with CL/F; **(C)**: HGB is correlated with CL/F; **(D)**: BUN is correlated with V/F; **(E)**: CREA is correlated with V/F; **(F)**: WBC is correlated with V/F. BUN: blood urea nitrogen; CL/F: apparent oral clearance; CREA: serum creatinine; HGB: Hemoglobin; V/F: apparent volume of distribution; WBC: white blood cell count.

Prior to screening for covariates, we assessed correlations among the covariates to mitigate issues related to multicollinearity. The covariates evaluated included the CYP3A5 genotype, CYP2C19 genotype, C_VRC_, and 26 other additional variables. By integrating the trend plots of parameters such as Ka, V/F, CL/F, along with the covariates, a graphical analysis was conducted (Figure 2). The analysis revealed that urea nitrogen was positively correlated with both CL/F and V/F, while CREA was similarly positively correlated with both CL/F and V/F. In contrast, white blood cell count demonstrated a negative correlation with V/F. Subsequently, C_VRC_, CYP2C19 genotype, and CYP3A5 genotype were included to develop the full covariate model. During the covariate selection process, a forward addition procedure was applied with criteria of *p* ≤ 0.01 and ΔOFV >6.635, followed by backward elimination with more stringent criteria of *p* < 0.001 and ΔOFV >10.828. The final covariates selected were C_VRC_ and CREA. A comparison results between the basic and final models are also presented in Table 3.

### 3.3 Model validation

#### 3.3.1 The plot of the goodness of fit

**FIGURE 3:**
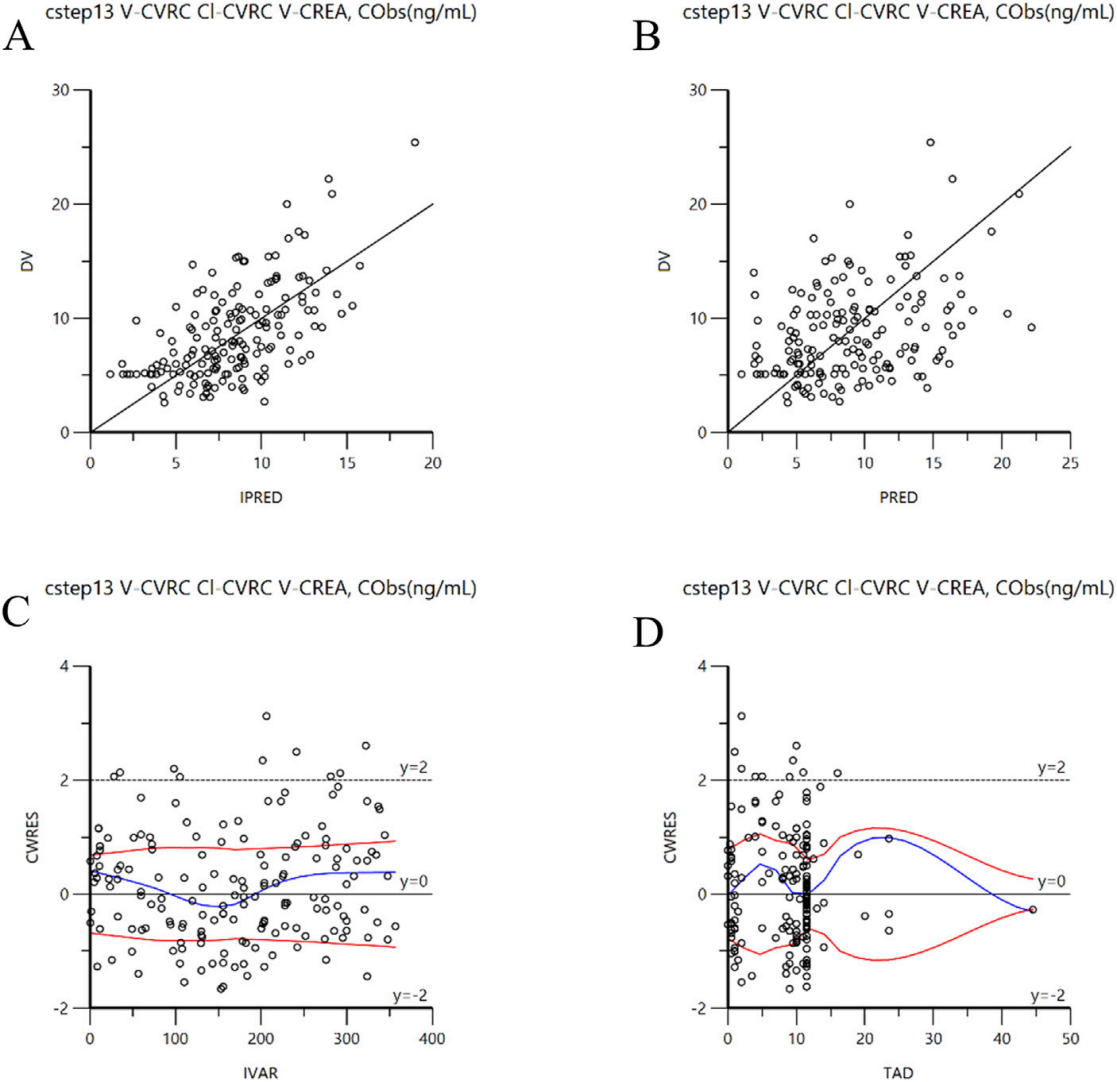
Goodness-of-fit plots for the final model. **(A)**: DV versus individual IPRED; **(B)**: DV versus PRED; **(C)**: CWRES versus IVAR; **(D)**: CWRES versus TAD. CWRES: Conditional population weighted residuals; DV: Dependent variable (observed concentration); IPRED: individual population prediction; PRED: population prediction; IVAR: individual observed value; TAD : Time after dose.

The goodness of fit plots of the final model are presented in Figures 3A–D. Figures 3A, B display the individual predicted values, population predicted value, and observed values, respectively. The concentration points are evenly distributed along the Y = X diagonal, indicating a strong correction between the predicted values (both individual and population) and the observed values in the final model. Figure 3C illustrates the distribution of conditionally weighted residuals (CWRES) against the individual predicted values, while Figure 3D shows the distribution of CWRES against the time after medication. The CWRES are centered around y = 0 and are uniformly distributed between y = ±2. Thus, the covariate model established is deemed reasonable.

#### 3.3.2 Bootstrap validation

The model was internally validated using the Bootstrap method with 1,000 resampling iterations. The mean values and 95% confidence intervals of pharmacokinetic parameters derived from the Bootstrap analysis are presented in Table 4. The average value of the parameter values obtained through the Bootstrap method are consistent with those of the final model, and the 95% confidence interval of the simulated results fall within a reasonable range, without including zero. Therefore, the parameter estimates from the final model are stable, reliable and minimally influenced by the sample distribution.

**TABLE 4:** Comparison of parameter estimates in the final model and bootstrap^a^.

Final model	Bootstrap results						
Parameter	Estimate	Mean	SD	CV%	Median	2.50%	97.50%
tvKa	8.39	8.388			8.39	8.39	8.39
tvV	2,690	2,655	59	22.39	2,630	1,480	4,060
tvCl	42.87	42.00	1.0	13.04	40.00	30	50
Θ_VRC-V_	−0.20	−0.20	0.08	−39.85	−0.20	−0.36	−0.03
Θ_VRC-CL_	−0.28	−0.34	0.24	−70.65	−0.28	−0.99	−0.21
Θ_CREA-V_	−0.40	−0.40	0.17	−41.55	−0.42	−0.72	−0.05
Interindividual variability							
ω^2^_V_	0.02	0.02	NA	6.67	NA	NA	NA
ω^2^_CL_	0.16	0.16	NA	6.32	NA	NA	NA
Residual variability							
σ	3.50	3.41	NA	5.39	3.42	3.06	3.74

^a^ CV: coefficient of variation; *Θ*_*VRC-V*_ *:* exponent for C_VRC_, as a covariate for V:*Θ*_*VRC-CL*_ *:* exponent for C_VRC_, as a covariate for CL; *Θ*_*CREA-V*_ *:* exponent for CREA, as a covariate for V; *ω:* inter-individual variation; σ: intraindividual variation; NA, not applicable.

#### 3.3.3 VPC validation

**FIGURE 4:**
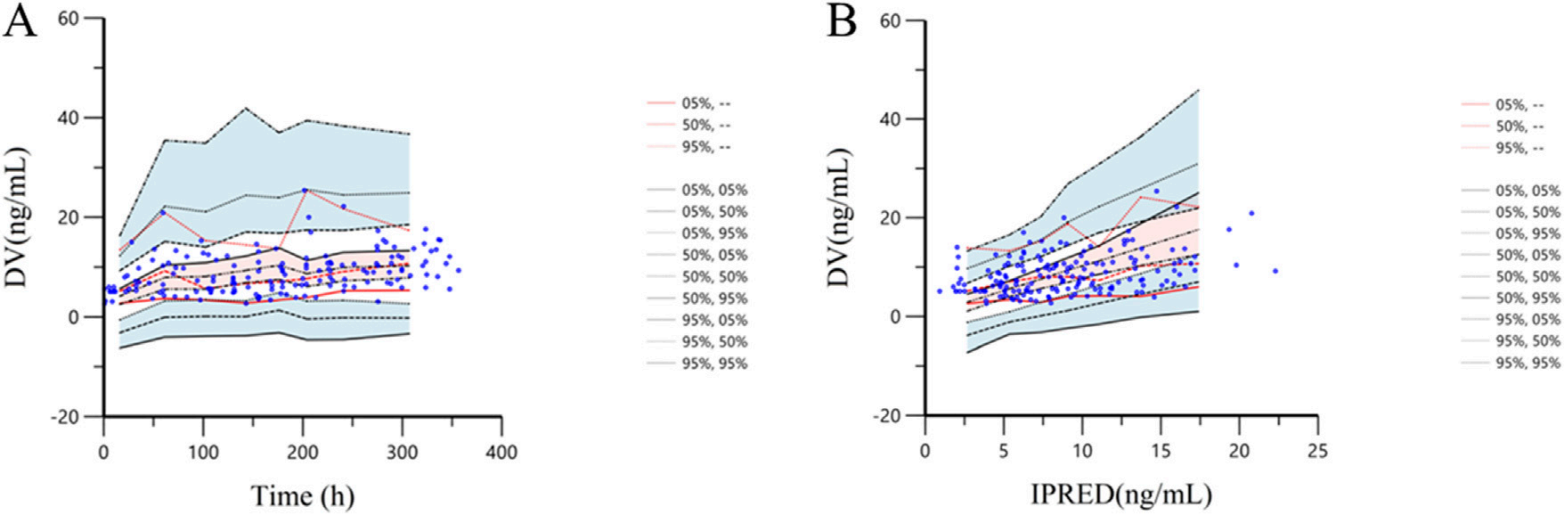
Prediction corrected-visual predictive check of tacrolimus final model. **(A)**: The *X*-axis represents time, while the *Y*-axis represents observed tacrolimus concentrations. Blue hollow dots represent the observed tacrolimus concentrations. Black dotted lines show the 5th, 50th and 95th percentiles of the simulated data. Solid red lines indicate the 5th, 50th and 95th percentiles of the observed data. The shaded areas represent the 90% CIs of the quantile corresponding to the simulated data. **(B)**: The *X*-axis represents IPRED, while the *Y*-axis represents observed tacrolimus concentrations. Blue hollow dots represent the observed tacrolimus concentrations. Black dotted lines show the 5th, 50th and 95th percentiles of the simulated data. Solid red lines indicate the 5th, 50th and 95th percentiles of the observed data. The shaded areas represent the 90% CIs of the quantile corresponding to the simulated data.

The VPC method was employed to conduct 1,000 simulation iterations to validate the final model. The VPC diagnosis plots are displayed in Figure 4A (TAD vs. DV) and Figure 4B (IVAR vs. DV). As shown in the figures, the 5th, 50th, and 95th quantiles of the observed values all fall within the 90% confidence interval of the corresponding predicted values. This indicates a high degree of agreement between the predicted and observed values, demonstrating the model’s strong predictive performance.

### 3.4 Monte Carlo simulation

#### 3.4.1 Monte Carlo simulations of different C_VRC_

**FIGURE 5:**
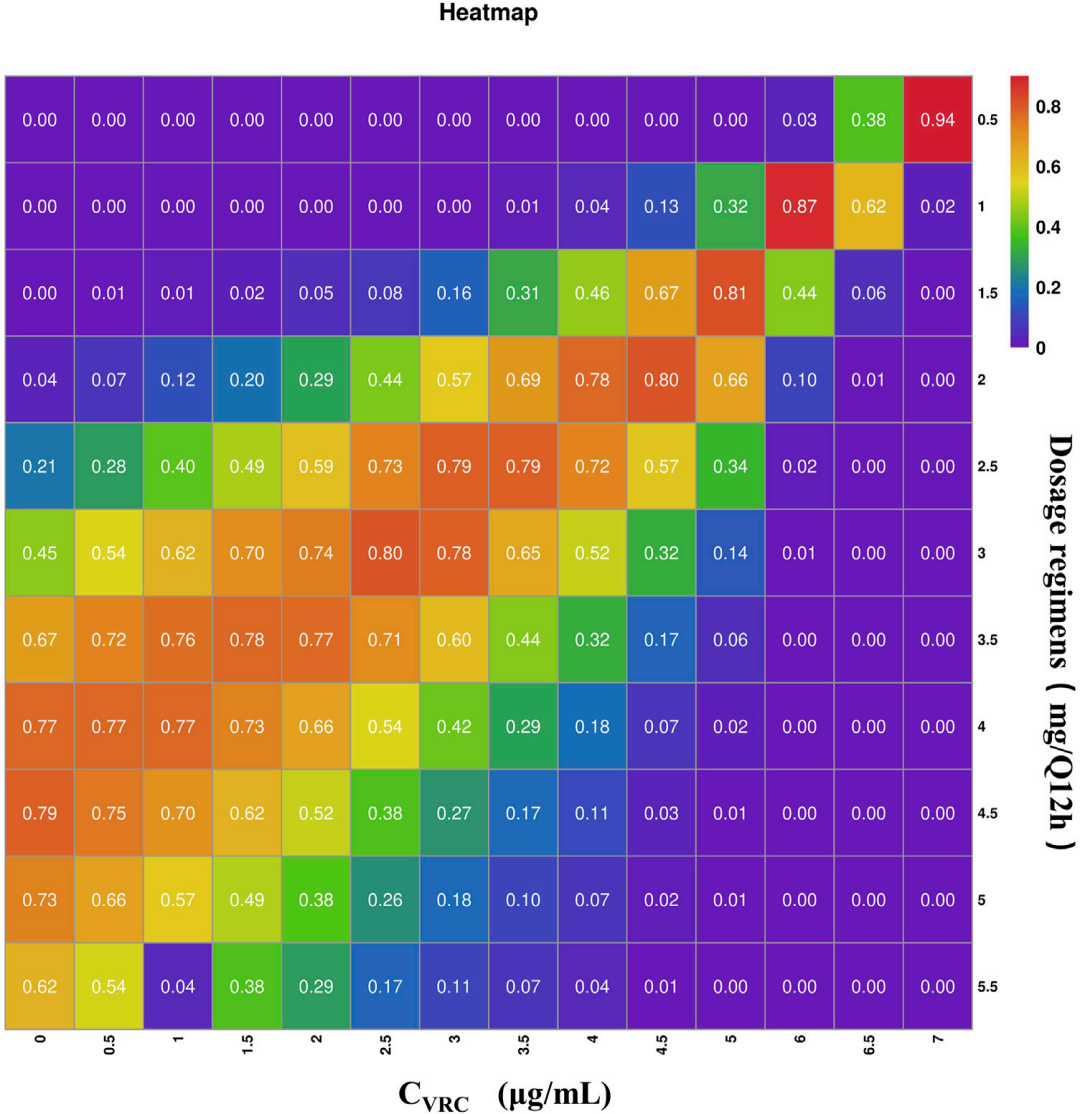
Heat map of tacrolimus PTA on day 3 under different administration regiments simulated based on different C_VRC_. The gradations of purple, blue-purple, blue, green, yellow, orange and red respectively represent the increasing of PTA from 0 to 100. The darker the red square is, the higher the PTA is; the darker the purple square is, the lower the PTA is.

Using the final established final PPK model, we identified C_VRC_ and CREA as two key covariates. We then evaluated 11 different tacrolimus dosing regimens and predicted tacrolimus trough concentration based on 15 different C_VRC_ levels (Table 5). For this analysis, we conducted 1,000 Monte Carlo simulations for patients with varying levels of C_VRC_ (ranging from 0 to 7.0 μg/mL). The CREA value, fixed at the median of 237 μmol/L, was held constant. The detailed mean and standard deviation of the trough concentration on the third day after tacrolimus administration are also presented in Table 5. The median tacrolimus trough concentrations based on the simulated data are visually depicted in Supplementary Figure S1. Based on the predicted tacrolimus concentrations, we further calculated the probability of target attainment (PTA) to maintain concentration within the therapeutic range of 5–10 ng/mL (Chen et al., 2022b). These results are provided in the (Supplementary Table S1). Additionally, a visual heat map of the data is shown in Figure 5.

**TABLE 5:** Tacrolimus trough concentration on day 3 based on different C_VRC_ and doses.

C_VRC_ (μg/mL	Tacrolimus dose (mg)^a^										
	0.5	1	1.5	2	2.5	3	3.5	4	4.5	5	5.5
	C_Tac_ (ng/mL)^b^										
**0**	0.82 (0.22)	1.65 (0.45)	2.47 (0.67)	3.29 (0.90)	4.12 (1.12)	4.94 (1.35)	5.76 (1.57)	6.59 (1.80)	7.41 (2.02)	8.23 (2.25)	9.06 (2.47)
**0.5**	0.88 (0.25)	1.75 (0.50)	2.63 (0.74)	3.50 (0.99)	4.38 (1.24)	5.25 (1.49)	6.13 (1.74)	7.00 (1.99)	7.88 (2.23)	8.76 (2.48)	9.63 (2.73)
**1**	0.95 (0.27)	1.89 (0.53)	2.84 (0.80)	3.79 (1.07)	4.73 (1.34)	5.68 (1.60)	6.62 (1.87)	7.57 (2.14)	8.52 (2.41)	9.46 (2.67)	10.41 (2.94)
**1.5**	1.01 (0.28)	2.02 (0.57)	3.03 (0.85)	4.05 (1.14)	5.06 (1.42)	6.07 (1.71)	7.08 (1.99)	8.09 (2.28)	9.10 (2.56)	10.12 (2.85)	11.13 (3.13)
**2**	1.10 (0.31)	2.20 (0.62)	3.30 (0.93)	4.40 (1.24)	5.49 (1.55)	6.59 (1.86)	7.69 (2.17)	8.79 (2.48)	9.89 (2.79)	10.99 (3.10)	12.09 (3.41)
**2.5**	1.21 (0.32)	2.42 (0.64)	3.63 (0.95)	4.84 (1.27)	6.05 (1.59)	7.26 (1.91)	8.47 (2.23)	9.68 (2.54)	10.89 (2.86)	12.10 (3.18)	13.31 (3.50)
**3**	1.32 (0.34)	2.65 (0.69)	3.97 (1.03)	5.29 (1.38)	6.62 (1.72)	7.94 (2.07)	9.27 (2.41)	10.59 (2.76)	11.91 (3.10)	13.24 (3.45)	14.56 (3.79)
**3.5**	1.49 (0.40)	2.98 (0.81)	4.46 (1.21)	5.95 (1.62)	7.44 (2.02)	8.93 (2.43)	10.41 (2.83)	11.90 (3.23)	13.39 (3.64)	14.88 (4.04)	16.37 (4.45)
**4**	1.65 (0.46)	3.31 (0.91)	4.96 (1.37)	6.61 (1.82)	8.26 (2.28)	9.92 (2.73)	11.57 (3.19)	13.22 (3.64)	14.88 (4.10)	16.53 (4.55)	18.18 (5.01)
**4.5**	1.92 (0.48)	3.83 (0.97)	5.75 (1.45)	7.66 (1.94)	9.58 (2.42)	11.49 (2.91)	13.41 (3.39)	15.33 (3.88)	17.24 (4.36)	19.16 (4.85)	21.07 (5.33)
**5**	2.26 (0.57)	4.51 (1.15)	6.77 (1.72)	9.02 (2.29)	11.28 (2.86)	13.54 (3.44)	15.79 (4.01)	18.05 (4.58)	20.30 (5.15)	22.56 (5.73)	24.82 (6.30)
**5.5**	2.73 (0.65)	5.47 (1.30)	8.20 (1.94)	10.93 (2.59)	13.67 (3.24)	16.40 (3.89)	19.14 (4.53)	21.87 (5.18)	24.60 (5.83)	27.34 (6.48)	30.07 (7.12)
**6**	3.47 (0.77)	6.94 (1.55)	10.40 (2.32)	13.87 (3.09)	17.34 (3.87)	20.81 (4.64)	24.27 (5.41)	27.74 (6.19)	31.21 (6.96)	34.68 (7.74)	38.14 (8.51)
**6.5**	4.76 (1.04)	9.53 (2.08)	14.29 (3.12)	19.05 (4.16)	23.82 (5.20)	28.58 (6.24)	33.34 (7.29)	38.10 (8.33)	42.87 (9.37)	47.63 (10.41)	52.39 (11.45)
**7**	7.31 (1.36)	14.61 (2.73)	21.92 (4.09)	29.22 (5.46)	36.53 (6.82)	43.83 (8.19)	51.14 (9.55)	58.44 (10.91)	65.75 (12.28)	73.06 (13.64)	80.36 (15.01)
***P***	<0.001	<0.001	<0.001	<0.001	<0.001	<0.001	<0.001	<0.001	<0.001	<0.001	<0.001

^a^ The frequency of administration is every 12 h.

^b^ mean (SD); C_VRC_, voriconazole concentration.

The PTA results indicate that when the C_VRC_ is 0 μg/mL, the recommended tacrolimus dose is 4 mg, with a dosing of 4–5.0 mg Q12 h achieving a standard tacrolimus concentration (5–10 ng/mL) in over 70% of cases. When the C_VRC_ is 5.0 μg/mL, the recommended tacrolimus dose decreased to 1.5 mg, with this dosage level achieving the target concentration (5–10 ng/mL) in 81.3% of the cases.

#### 3.4.2 Monte Carlo simulations with different CREA values

Given that CREA was identified as a significant covariate in this model, we performed 1,000 Monte Carlo simulations based on 11 tacrolimus dosing regimens across 10 different CREA levels. In this analysis, C_VRC_ was fixed at to 0 μmol/L to eliminate the influence of voriconazole use. The simulation results are illustrated in Table 6.

**TABLE 6:** Tacrolimus trough concentration on day 3 based on different CREA and doses.

Dose^a^	CREA (μmol/L)^b^										
(mg/q12 h)	40	100	160	400	600	800	1,000	1,600	1800	2000	*P*
**0.25**	0.59 (0.12)	0.78 (0.18)	0.88 (0.21)	1.13 (0.31)	1.21 (0.34)	1.29 (0.40)	1.31 (0.42)	1.41 (0.48)	1.45 (0.49)	1.42 (0.49)	<0.001
**0.5**	0.59 (0.12)	0.78 (0.18)	0.88 (0.21)	1.13 (0.31)	1.21 (0.34)	1.29 (0.40)	1.31 (0.42)	1.41 (0.48)	1.45 (0.49)	1.42 (0.49)	<0.001
**1**	1.17 (0.24)	1.56 (0.35)	1.77 (0.43)	2.26 (0.62)	2.42 (0.68)	2.57 (0.79)	2.63 (0.84)	2.81 (0.97)	2.90 (0.99)	2.84 (0.98)	<0.001
**1.5**	1.76 (0.36)	2.34 (0.53)	2.65 (0.64)	3.39 (0.93)	3.63 (1.02)	3.86 (1.19)	3.94 (1.26)	4.22 (1.45)	4.35 (1.48)	4.26 (1.47)	<0.001
**2**	2.35 (0.48)	3.12 (0.70)	3.54 (0.85)	4.52 (1.24)	4.84 (1.36)	5.15 (1.58)	5.25 (1.68)	5.62 (1.94)	5.79 (1.97)	5.67 (1.96)	<0.001
**2.5**	2.94 (0.60)	3.90 (0.88)	4.42 (1.07)	5.65 (1.55)	6.05 (1.70)	6.43 (1.98)	6.56 (2.09)	7.03 (2.42)	7.24 (2.46)	7.09 (2.45)	<0.001
**3**	3.52 (0.73)	4.68 (1.05)	5.31 (1.28)	6.78 (1.86)	7.27 (2.04)	7.72 (2.37)	7.88 (2.51)	8.43 (2.91)	8.69 (2.96)	8.51 (2.94)	<0.001
**3.5**	4.11 (0.85)	5.46 (1.23)	6.19 (1.49)	7.91 (2.16)	8.48 (2.38)	9.01 (2.77)	9.19 (2.93)	9.84 (3.39)	10.14 (3.45)	9.93 (3.43)	<0.001
**4**	4.70 (0.97)	6.24 (1.41)	7.08 (1.70)	9.03 (2.47)	9.69 (2.72)	10.30 (3.16)	10.50 (3.35)	11.25 (3.88)	11.59 (3.94)	11.35 (3.92)	<0.001
**4.5**	5.29 (1.09)	7.02 (1.58)	7.96 (1.92)	10.16 (2.78)	10.90 (3.06)	11.58 (3.56)	11.82 (3.77)	12.65 (4.36)	13.04 (4.43)	12.77 (4.41)	<0.001
**5**	5.87 (1.21)	7.80 (1.76)	8.85 (2.13)	11.29 (3.09)	12.11 (3.40)	12.87 (3.95)	13.13 (4.19)	14.06 (4.84)	14.49 (4.93)	14.19 (4.90)	<0.001
**5.5**	6.46 (1.33)	8.58 (1.93)	9.73 (2.34)	12.42 (3.40)	13.32 (3.74)	14.16 (4.35)	14.44 (4.61)	15.46 (5.33)	15.94 (5.42)	15.60 (5.39)	<0.001

^a^ The frequency of administration is every 12 h.

^b^ mean (SD); CREA, serum creatinine.

**FIGURE 6:**
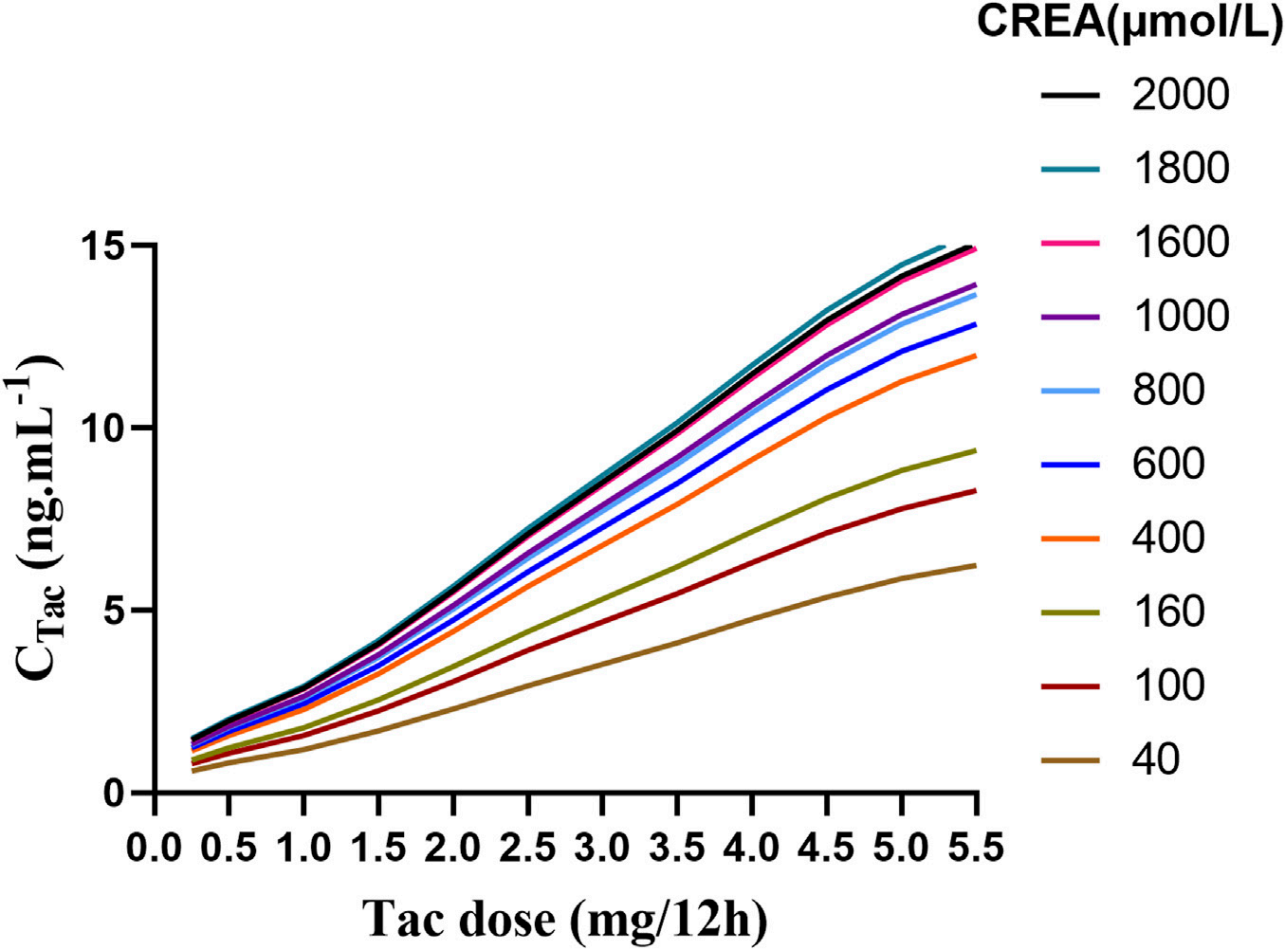
The fitting curve of tacrolimus trough concentration on the third day was simulated based on different creatinine values.

The result indicated that, under the same dosing regimen, higher CREA were associated with higher tacrolimus concentration (Figure 6). The PTA for tacrolimus trough concentrations on the third day was also calculated., with the results provided in Supplementary Table S2. The predicted tacrolimus concentration and their corresponding PTA values for each CREA level were used. The predicted tacrolimus concentration and their corresponding PTA values for each CREA level were used to create visual heat maps, which in Supplementary Figures S2, S3. Based on these results, an appropriate tacrolimus dose can be determined. For example, when the CREA concentration is 40 μmol/L, the recommended tacrolimus dose is 5–5.5 mg Q12 h, achieving a PTA above 70%.

#### 3.4.3 Practical application

The Monte Carlo simulation results provide robust, evidence-based guidelines for optimizing tacrolimus dosing in renal transplant recipients co-administered with voriconazole. To facilitate the clinical application of these findings, we have developed (Supplementary Table S3), which consolidates the recommended tacrolimus dosing regimens across a spectrum of C_VRC_ and CREA levels. By thoroughly evaluating the pharmacokinetic interactions influenced by C_VRC_ and CREA, we delineated dosing regimens to ensure the achievement of therapeutic drug levels with a high probability of target attainment. The analysis reveals that as C_VRC_ increases, the required tacrolimus dose must be correspondingly reduced to mitigate the risk of drug toxicity. Conversely, elevated CREA levels, indicative of diminished renal function, necessitate a decrease in tacrolimus dosing to maintain therapeutic efficacy while minimizing potential adverse effects.

## 4 Discussion

In this study, we developed a one-compartment model with first-order elimination to describe the PPK of tacrolimus in RTRs undergoing voriconazole co-therapy. This model, applied for the first time in such a context, provides accurate predictions of tacrolimus concentration across various C_VRC_ levels. According to tacrolimus prescribing information (2015),peak blood concentration are typically reached within 1–3 h after oral administration, with an average oral bioavailability of 20%–25%. Most patients achieve steady-state concentrations within 3 days. The steady-state distribution volume of tacrolimus in healthy individuals is approximately 1,300 L, based on the whole blood concentrations. However, in transplant patients, the half-life of tacrolimus is significantly shorter, and the clearance rate is considerably higher compared to healthy subjects. Tacrolimus is primarily metabolized in the liver, with CYP450-3A4 as the principal metabolic enzyme (Iwamoto et al., 2015). What sets this study apart from previous research is our ability to recommend specific tacrolimus doses for different levels of voriconazole, based on simulation results of tacrolimus trough concentration and the PTA. To our knowledge, no prior studies have adjusted tacrolimus dosing based on C_VRC_ levels, making this approach a novel contribution to the field.

Meanwhile, the population’s typical V/F, derived from plasma concentration data, was estimated at 2690 L, a value notably higher than that observed in healthy individuals. In the final model, voriconazole concentration and CREA emerged as critical covariates. As voriconazole concentration increased, both V/F and CL/F decreased. Similarly, higher CREA levels were associated with a further reduction in V/F. Our findings regarding the influence of co-administration with voriconazole on the distribution of tacrolimus are consistent with previous studies. Voriconazole is known to inhibit the CYP3A4 enzyme, which plays a crucial role in the metabolism of tacrolimus (Fujita et al., 2013; Imamura et al., 2016; Gong et al., 2023). Polymorphisms in CYP3A5, POR, and CYP2C19 are also important biomarkers for individualized tacrolimus dosage adjustments (Suetsugu et al., 2019). This inhibition can increased systemic exposure to tacrolimus, leading to alterations in its distribution throughout the body. Furthermore, the interaction between voriconazole and tacrolimus at the level of drug transporters, such as P-glycoprotein (P-gp), can further impact the distribution of tacrolimus (Fu et al., 2019). Additionally, changes in protein binding due to the co-administration of voriconazole may contribute to modifications in tacrolimus distribution (Yuan et al., 2020). Lastly, the impact of voriconazole on liver and kidney function can indirectly influence tacrolimus distribution (Theuretzbacher et al., 2006; Neofytos et al., 2012). In a related study, Staatz and Tett (2002) collected 4,527 tacrolimus blood samples from 337 kidney transplant recipients and developed a two-compartment model. They reported that lower CREA levels were associated with increased tacrolimus clearance. Cheng et al. (2023) also found that direct bilirubin, albumin, and estimated glomerular filtration rate (eGFR) were significant factors influencing voriconazole trough concentrations and suggested that eGFR and platelet count should also be considered when administering voriconazole. Besides, Jahan et al. (2015) explored the clinical efficacy and pharmacokinetics of tacrolimus in children with steroid-resistant nephrotic syndrome and found that patients with elevated CREA may have lower trough concentration and AUC_0–12 h_. These studies collectively demonstrate that renal function indicators such as CREA and eGFR can significantly affect the concentration of tacrolimus and voriconazole.

In addition, we compared the parameters of the model established in this study with those of the tacrolimus PPK models from other studies. The summary of the models and their parameters is provided in Table 7. The comparison results revealed that most studies utilized either one-compartment or two-compartment models, indicating that the one-compartment model used in our study was also reasonable. Meanwhile, in our study, the CL/F was estimated 42.87 L/h, which is significantly reduced compared to other RTRs and healthy volunteers. This value is approximately 1/3 of that observed in patients not receiving voriconazole co-therapy. Beyond voriconazole, other factors identified as covariables include hematocrit, weight, Wu-Zhi capsule usage, and CYP3A4, and CYP3A5 genotypes (Barry and Levine, 2010; Bergmann et al., 2014; Jacobo-Cabral et al., 2015; Billing et al., 2017; Andrews et al., 2018; Campagne et al., 2018; Andrews et al., 2019).

**TABLE 7:** Parameter comparison of population pharmacokinetic models^a^.

Data sources	Population	Model	Covariate	Parameter
Result of this study	Renal transplantation, adult	One compartment	C_VRC_, CREA	Ka = 8.39/h CL/F = 42.87L/h V/F = 2690 L
Anders et al. (Andrews et al., 2018)	Renal transplantation, children	Two compartment	weight, Glomerular Filtration Rate, Hematocrit and CYP3A5	Ka = 0.56/h CL/F = 26.7 L/h Q/F = 114L/h V1/F = 206 L V2/F = 1520 L
Anders et al. (Andrews et al., 2019)	Renal transplantation, adult	Two compartment	CYP3A5, CYP3A4*1, BSA, CREA, age, albumin, and hematocrit	Ka = 3.6/h CL/F = 23.0 L/h Q/F = 79.6L/h V1/F = 692 L V2/F = 5340 L
Ogasawara et al. (Ogasawara et al., 2013)	Renal transplantation, adult	Two compartment	CYP3A5, MRP2	Ka = 0.544 h CL/F = 20.7 L/h Q/F = 70.7 L/h V1/F = 234 L V2/F = 1319 L
Lu et al. (Lu et al., 2015)	Healthy volunteers and liver transplant patients, adult	Two compartment	Population, ALT	Ka = 0.419 h CL/F = 32.8 L/h Q/F = 76.3 L/h V1/F = 22.7 L V2/F = 916 L
Xiao et al. (Chen et al., 2021b)	Hematopoietic stem cell transplantation, Children	One compartment	weight, voriconazole use use	Ka = 4.48/h CL/F = 35.4 L/h V/F = 5970 L
Cai et al. (Cai et al., 2020)	Lung transplantation, adult	One compartment	Hematocrit, POT, tacrolimus daily dose, voriconazole use、CYP3A5	Ka = 13.1/h CL/F = 35.4 L/h V/F = 5970 L

^a^ ALT, alanine aminotransferase; BSA, body surface area; CREA, serum creatine; CYP, cytochrome P450; CL/F, apparent oral clearance; Ka, absorption rate constant; MRP2, multidrug resistance-associated protein 2; POT, postoperative time; Q/F, apparent inter-compartmental clearance; V1/F, apparent central volume of distribution after oral administration; V2/F, apparent peripheral volume of distribution after oral administration.

Therefore, different studies may include various covariates due to differing sample sizes and study designs. In our study, only C_VRC_ and CREA were identified as significant covariates, likely due to the limited sample size, which may have restricted the inclusion of other potential covariates such as hematocrit, CYP3A5, and CYP2C19 genotypes. Additionally, we did not collect CYP3A4 genotype information, which could have further influenced the model.

Besides, CYP3A5 polymorphisms are known to significantly affect tacrolimus pharmacokinetics. Patients expressing the CYP3A5*1*3 genotype typically have a higher clearance rate and therefore require higher daily doses to achieve therapeutic drug levels compared to non-expressers (CYP3A5*3*3). Anders et al. found that pharmacokinetic parameters of CYP3A5 differ across metabolic types, with patients expressing CYP3A5 having a higher clearance rate than those without CYP3A5 expression (Andrews et al., 2018). This finding is supported by Ferraris et al., who demonstrated that patients with the CYP3A5*1*3 genotype had lower dose-adjusted tacrolimus trough levels and required higher daily doses to achieve therapeutic drug levels, underscoring the need for individualized dosing based on genotype (Ferraris et al., 2011). Similarly, Ogasawara et al. also identified that CYP3A5 was also a significant covariate for the apparent clearance of tacrolimus (Ogasawara et al., 2013). Additionally, the meta-analysis by Lee et al. (2022), further supported the association between CYP3A5 expression and tacrolimus pharmacokinetics, particularly in patients carrying the POR28 allele. These findings underscore the importance of considering CYP3A5 polymorphisms when determining optimal tacrolimus dosing regimens (Lee et al., 2022). However, patients with the CYP3A5*1*1 genotype were not included in this study, which may explain why the CYP3A5 genotype was not a covariate in our final model. It is also possible that the effect of C_VRC_ was more pronounced than other factors, leading to the inclusion of only the most influential covariates in the final model. Several other studies have demonstrated that postoperative time can significantly affect the pharmacokinetic parameters of tacrolimus (Wang et al., 2019; Gong et al., 2020; Chen L. et al., 2021a; Srinivas et al., 2021). But in this study, postoperative time did not emerge as a key covariable influencing tacrolimus metabolism. Campagne et al. (2018) analyzed 63 studies on nonlinear mixed-effects models of tacrolimus published in the past 20 years and found that most studies focused on adult and pediatric renal and liver transplantation, and more than 50% of the PPK studies used one-compartment model and two-compartment model with delayed absorption. However, there are differences in the pharmacokinetics of tacrolimus in different populations, so studying the pharmacokinetic parameters in different drug combinations is necessary.

Although this study is the first to establish a PPK model for tacrolimus in RTRs co-administered with voriconazole, allowing us to predict tacrolimus concentration and estimate optimal dosage based on different C_VRC_ levels, there are still some limitations. First, several studies have shown that postoperative time can significantly affect the pharmacokinetic parameters of tacrolimus. However, the data collected in this study were from RTRs within 15 days post-surgery. As a result, the decision to use a one-compartment model rather than a more complex two-compartment model may have been influenced by the limited amount of data available. The absence of a biphasic or more complex concentration-time curve pattern could be attributed to the limited sampling points, which could have hindered our ability to fully capture the true underlying pharmacokinetic characteristics. Second, the limited sample size in this study meant that only internal verification of the model was possible, without the benefit of external validation. Additionally, our reliance on a narrow range of drug dosage regimens for the simulations represents a further limitation. Consequently, to derive more accurate and optimal tacrolimus dosing regimens, it would be necessary to conduct a greater number of Monte Carlo simulations across a broader range of scenarios. Expanding the scope of these simulations would provide a more comprehensive understanding of the PTA across a wider spectrum of dosing strategies.

Therefore, further studies should focus on external verification of the model and clinical verification of these findings. Third, while we successfully established a stable PPK model, it is still important to note that most of the tacrolimus concentrations data collected were trough levels, with minimal clinical intervention. Additionally, the Ka value was fixed during the model’s development, which restricted our ability to explore the impact of other covariates on Ka. The simulations conducted to evaluate the model’s predictive performance, taking into account the VPC results (Figure 4A) and the residual variability indicated by the sigma value in Table 4, suggest that factors beyond voriconazole concentration and CREA may contribute to the observed inter- and intra-individual variability in tacrolimus pharmacokinetics within this population. Furthermore, the high CV% associated with the Θ_VRC-CL_ parameter highlights potential uncertainty in its estimation, underscoring the need for additional research to identify and integrate other significant covariates. Despite these limitations, the findings of this study offer valuable insights that may contribute to the development of more robust and reliable models for predicting tacrolimus concentrations, ultimately aiding clinicians in optimizing patient outcomes. Further research should aim to address the identified limitations, thereby enhancing the precision and applicability of PPK models in clinical settings.

In healthy subjects, the rate and extent of tacrolimus absorption are highest when on an empty stomach (2015). Diet can reduce both the absorption rate and extent of tacrolimus, with this effect being most pronounced after consuming high-fat foods. Since dietary information was not collected in this study, the potential impact of food on tacrolimus metabolism was not considered. Therefore, prospective studies with large sample sizes and multicenter designs are necessary for further validation and exploration. In this study, we utilized a mixed linear function model that incorporated both additive and multiplicative covariate structures. However, there may be limitations in accurately estimating high voriconazole concentrations. The range of voriconazole concentrations included in our study was limited to 0–3.38 μg/mL. As a result, extrapolating the model predictions beyond this range, particularly for high concentrations, may lead to less accurate estimates. This limitation should be considered when interpreting the results of our study.

## 5 Conclusion

The population pharmacokinetics of tacrolimus co-administered with 15 days after renal transplantation were effectively described using the models presented in this study. The final model identified C_VRC_ and CREA as critical covariates. Patients with higher C_VRC_ had lower tacrolimus CL/F and V/F, while higher CREA also led to a reduction in tacrolimus CL/F. Based on different C_VRC_ and CREA, clinicians can predict tacrolimus concentrations and adjust the dosage accordingly. In addition, the Monte Carlo simulation results offer clear, actionable dosage recommendations tailored to C_VRC_ and CREA values. In general, a relatively lower dosage of tacrolimus is required as C_VRC_ increases. Moreover, the influence of voriconazole on tacrolimus concentration was found to be more significant than that of CREA.

## Data Availability

The raw data supporting the conclusions of this article will be made available by the authors, without undue reservation.
