# Supplementary material for: Individualized dosing parameters for tacrolimus in the presence of voriconazole: a real-world PopPK study
Source: Front Pharmacol. 2024 Sep 10;15:1439232. doi: 10.3389/fphar.2024.1439232 (PMC11419969; doi:10.3389/fphar.2024.1439232)
Supplement: Supplementary file 1 [file DataSheet1.docx]

Supplementary Material

How to predict tacrolimus dose and concentration during voriconazole co-therapy in renal transplantation recipients?

**Yi-Chang Zhao^1,2^, Chen-Lin Xiao^1,2^, Jing-Jing Hou ^1,2^, Jia-Kai Li ^1,2^, Bi-Kui Zhang^1,2^, Xu-Biao Xie^3^, Chun-Hua Fang^3^, Indy Sandaradura^4,5^, Feng-Hua Peng^3^*and Miao Yan ^1,2,^***

*** Correspondence:** Miao Yan([yanmiao@csu.edu.cn](mailto:yanmiao@csu.edu.cn)) & Feng-Hua Peng([pfh3327@csu.edu.cn](mailto:pfh3327@csu.edu.cn))

# Supplementary Figures and Tables

## Supplementary Figures

**
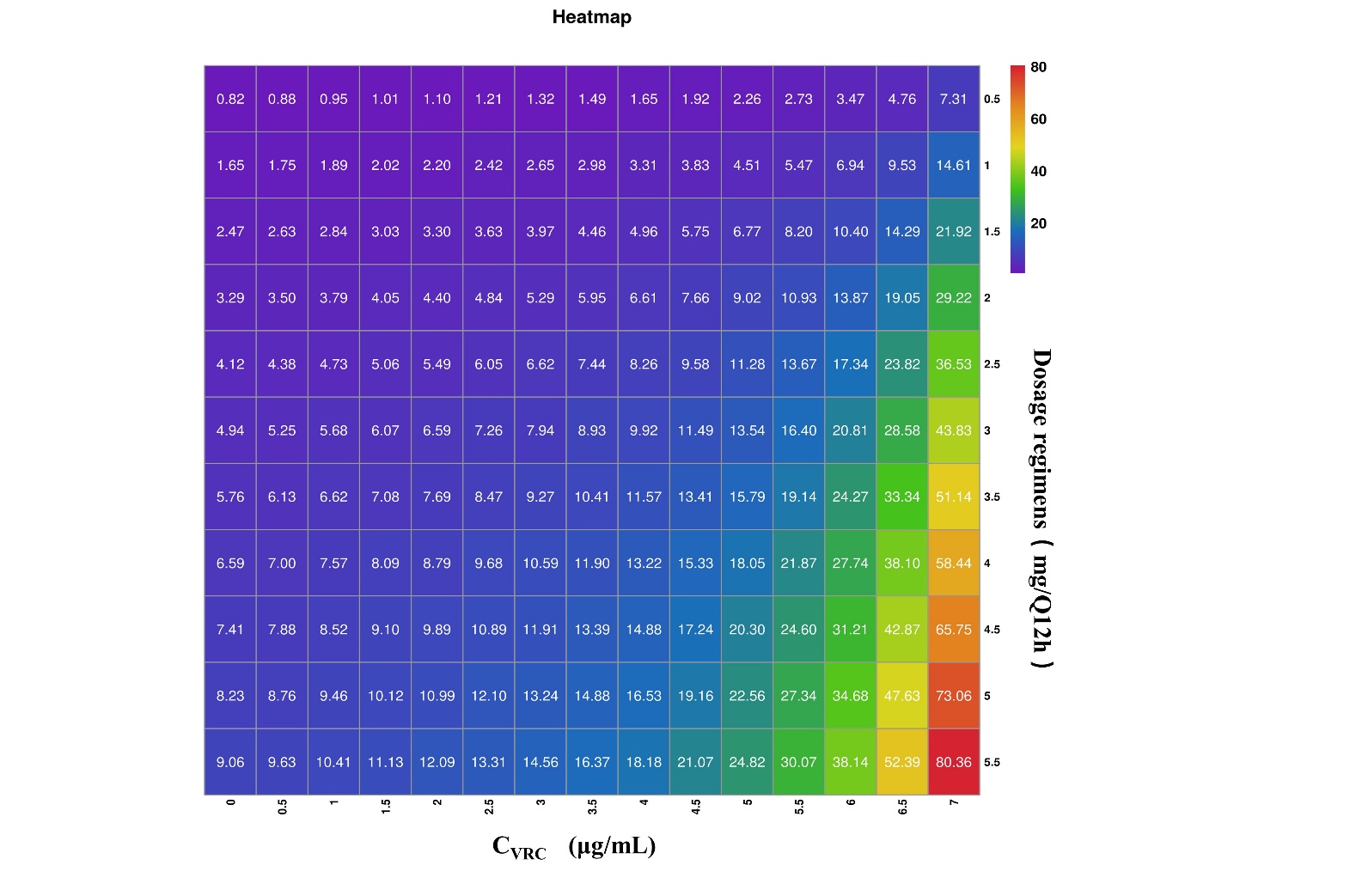
**

**Supplementary Figure 1.** Heat map of predicted tacrolimus trough concentration on day 3 under different administration regiments simulated based on different C_VRC_. The gradations of purple, blue-purple, blue, green, yellow, orange and red respectively represent the increasing of tacrolimus concentration. The darker the red square is, the higher the concentration is; the darker the purple square is, the lower the concentration is.

**
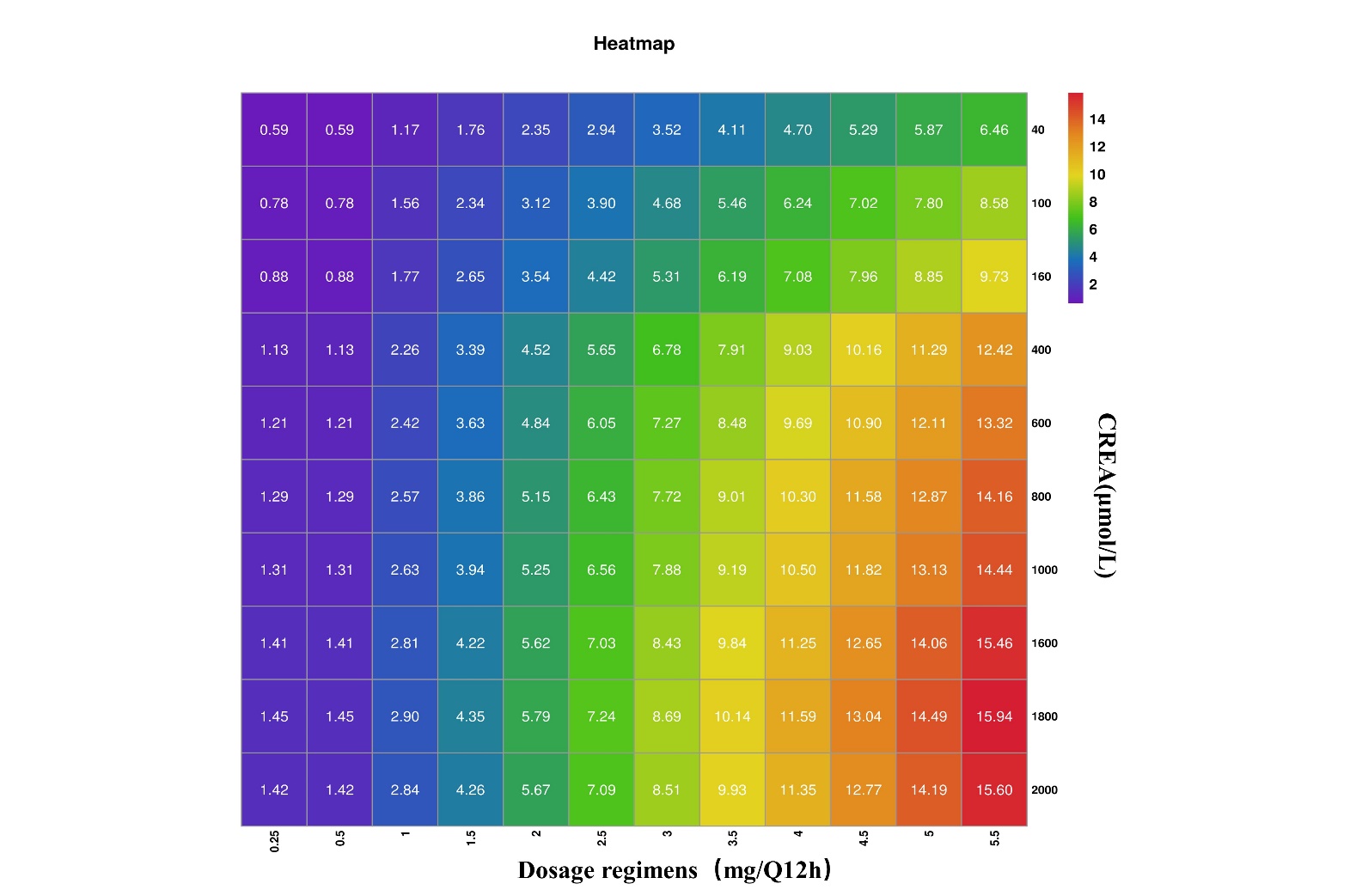
**

**Supplementary Figure 2.** Heat map of predicted tacrolimus trough concentration on day 3 under different administration regiments simulated based on different CREA. The gradations of purple, blue-purple, blue, green, yellow, orange and red respectively represent the increasing of tacrolimus concentration. The darker the red square is, the higher the concentration is; the darker the purple square is, the lower the concentration is.

**
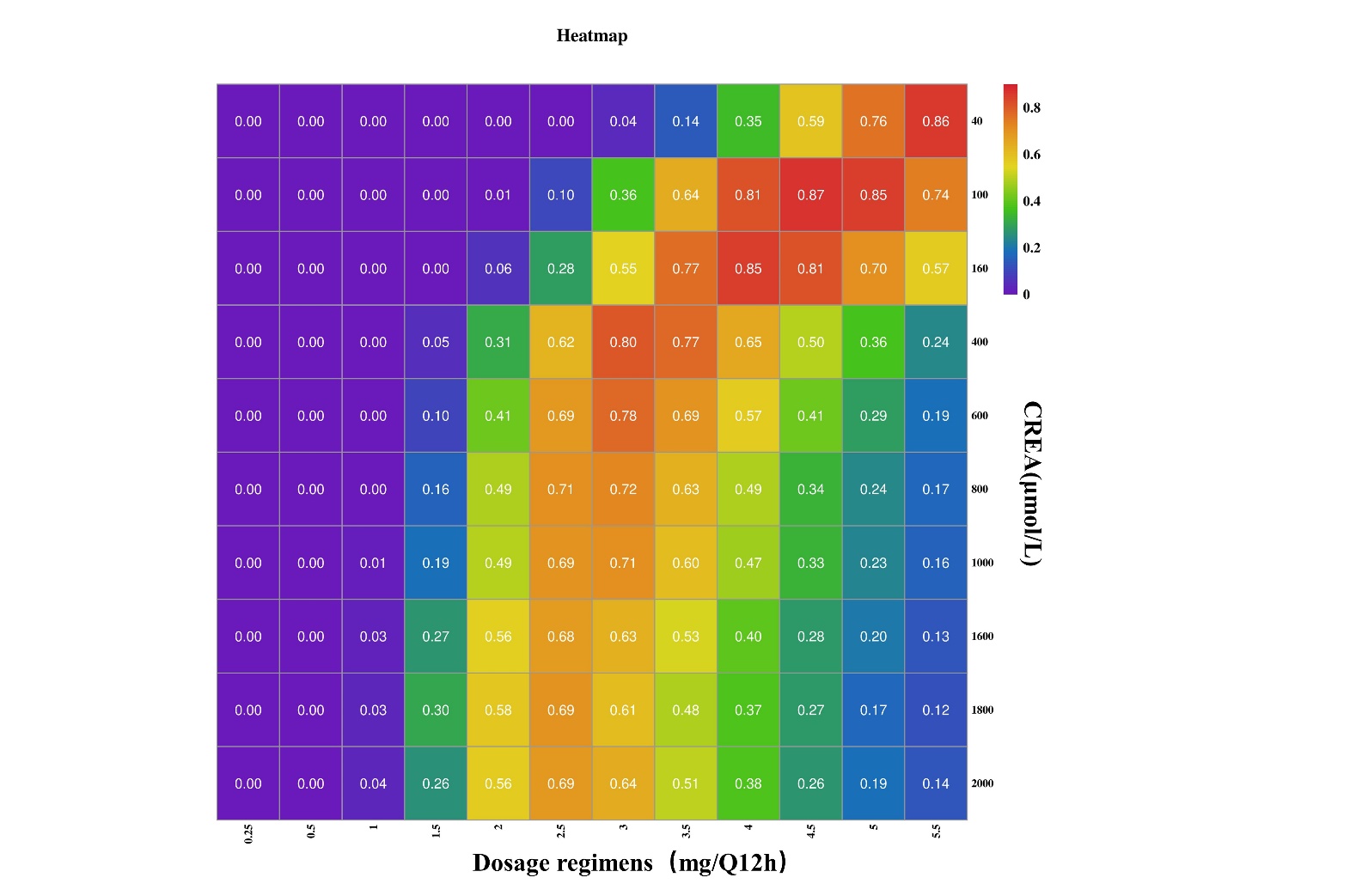
**

**Supplementary Figure 3.** Heat map of tacrolimus PTA on day 3 under different administration regiments simulated based on different CREA. The gradations of purple, blue-purple, blue, green, yellow, orange and red respectively represent the increasing of PTA from 0 to 100. The darker the red square is, the higher the PTA is; the darker the purple square is, the lower the PTA is.

## Supplementary Tables

# Table S1. The PTA on day 3 based on different C_VRC_ and doses.

| C_VRC_**(μg/mL)** | | | | | | | | | | | | | | | | | | |
| --- | --- | --- | --- | --- | --- | --- | --- | --- | --- | --- | --- | --- | --- | --- | --- | --- | --- | --- |
| **Dose^a^**  **(q12h）** | **Level** | **0** | **0.5** | **1** | **1.5** | **2** | **2.5** | **3** | **3.5** | **4** | **4.5** | **5** | **6** | **6.5** | **7** | ***P*** |  |  |
|  |  | **PTA(%)** | | | | | | | | | | | | | | | |  |
| 0.5 | <5.0 | 100.00 | 100.00 | 100.00 | 100.00 | 100.00 | 100.00 | 100.00 | 100.00 | 100.00 | 100.00 | 100.00 | 97.00 | 62.40 | 2.20 | <0.001 |  |  |
|  | 5.0-10.0 | 0.00 | 0.00 | 0.00 | 0.00 | 0.00 | 0.00 | 0.00 | 0.00 | 0.00 | 0.00 | 0.00 | 3.00 | 37.60 | 93.80 |  |  |  |
|  | >10.0 | 0.00 | 0.00 | 0.00 | 0.00 | 0.00 | 0.00 | 0.00 | 0.00 | 0.00 | 0.00 | 0.00 | 0.00 | 0.00 | 4.00 |  |  |  |
| 1.0 | <5.0 | 100.00 | 100.00 | 100.00 | 100.00 | 100.00 | 100.00 | 99.70 | 98.80 | 96.10 | 87.30 | 68.40 | 9.60 | 0.80 | 0.00 | <0.001 |  |  |
|  | 5.0-10.0 | 0.00 | 0.00 | 0.00 | 0.00 | 0.00 | 0.00 | 0.30 | 1.20 | 3.90 | 12.70 | 31.60 | 87.40 | 61.60 | 2.20 |  |  |  |
|  | >10.0 | 0.00 | 0.00 | 0.00 | 0.00 | 0.00 | 0.00 | 0.00 | 0.00 | 0.00 | 0.00 | 0.00 | 3.00 | 37.60 | 97.80 |  |  |  |
| 1.5 | <5.0 | 100.00 | 99.50 | 98.60 | 98.00 | 95.10 | 92.10 | 84.30 | 69.00 | 53.80 | 32.70 | 14.20 | 0.90 | 0.00 | 0.00 | <0.001 |  |  |
|  | 5.0-10.0 | 0.00 | 0.50 | 1.40 | 2.00 | 4.90 | 7.90 | 15.70 | 31.00 | 46.10 | 67.20 | 81.30 | 43.70 | 6.20 | 0.00 |  |  |  |
|  | >10.0 | 0.00 | 0.00 | 0.00 | 0.00 | 0.00 | 0.00 | 0.00 | 0.00 | 0.10 | 0.10 | 4.50 | 55.40 | 93.80 | 100.00 |  |  |  |
| 2.0 | <5.0 | 96.40 | 92.80 | 87.80 | 80.40 | 70.50 | 56.40 | 42.90 | 30.10 | 18.60 | 7.20 | 2.40 | 0.00 | 0.00 | 0.00 | <0.001 |  |  |
|  | 5.0-10.0 | 3.60 | 7.20 | 12.20 | 19.60 | 29.50 | 43.60 | 56.80 | 68.70 | 77.50 | 80.10 | 66.00 | 9.60 | 0.80 | 0.00 |  |  |  |
|  | >10.0 | 0.00 | 0.00 | 0.00 | 0.00 | 0.00 | 0.00 | 0.30 | 1.20 | 3.90 | 12.70 | 31.60 | 90.40 | 99.20 | 100.00 |  |  |  |
| 2.5 | <5.0 | 79.30 | 71.70 | 60.30 | 51.20 | 40.10 | 26.10 | 17.90 | 10.50 | 7.00 | 1.60 | 0.50 | 0.00 | 0.00 | 0.00 | <0.001 |  |  |
|  | 5.0-10.0 | 20.70 | 28.30 | 39.70 | 48.60 | 59.50 | 72.70 | 79.00 | 78.50 | 72.20 | 57.50 | 33.90 | 1.80 | 0.10 | 0.00 |  |  |  |
|  | >10.0 | 0.00 | 0.00 | 0.00 | 0.20 | 0.40 | 1.20 | 3.10 | 11.00 | 20.80 | 40.90 | 65.60 | 98.20 | 99.90 | 100.00 |  |  |  |
| 3.0 | <5.0 | 54.60 | 45.20 | 36.10 | 27.80 | 20.70 | 12.00 | 6.10 | 3.70 | 2.00 | 0.40 | 0.20 | 0.00 | 0.00 | 0.00 | <0.001 |  |  |
|  | 5.0-10.0 | 45.40 | 54.30 | 62.50 | 70.20 | 74.40 | 80.10 | 78.20 | 65.30 | 51.80 | 32.30 | 14.00 | 0.90 | 0.00 | 0.00 |  |  |  |
|  | >10.0 | 0.00 | 0.50 | 1.40 | 2.00 | 4.90 | 7.90 | 15.70 | 31.00 | 46.20 | 67.30 | 85.80 | 99.10 | 100.00 | 100.00 |  |  |  |
| 3.5 | <5.0 | 32.60 | 25.60 | 19.60 | 14.10 | 9.00 | 4.90 | 3.10 | 1.50 | 0.40 | 0.10 | 0.00 | 0.00 | 0.00 | 0.00 | <0.001 |  |  |
|  | 5.0-10.0 | 66.60 | 72.20 | 75.60 | 77.90 | 77.20 | 71.40 | 60.40 | 44.40 | 32.10 | 16.80 | 6.00 | 0.20 | 0.00 | 0.00 |  |  |  |
|  | >10.0 | 0.80 | 2.20 | 4.80 | 8.00 | 13.80 | 23.70 | 36.50 | 54.10 | 67.50 | 83.10 | 94.00 | 99.80 | 100.00 | 100.00 |  |  |  |
| 4.0 | <5.0 | 19.10 | 15.40 | 10.40 | 7.20 | 4.70 | 2.60 | 1.20 | 0.90 | 0.10 | 0.00 | 0.00 | 0.00 | 0.00 | 0.00 | <0.001 |  |  |
|  | 5.0-10.0 | 77.30 | 77.40 | 77.40 | 73.20 | 65.80 | 53.80 | 41.70 | 29.20 | 18.50 | 7.20 | 2.40 | 0.00 | 0.00 | 0.00 |  |  |  |
|  | >10.0 | 3.60 | 7.20 | 12.20 | 19.60 | 29.50 | 43.60 | 57.10 | 69.90 | 81.40 | 92.80 | 97.60 | 100.00 | 100.00 | 100.00 |  |  |  |
| 4.5 | <5.0 | 10.60 | 8.30 | 6.00 | 4.30 | 2.70 | 1.00 | 0.60 | 0.30 | 0.10 | 0.00 | 0.00 | 0.00 | 0.00 | 0.00 | <0.001 |  |  |
|  | 5.0-10.0 | 78.70 | 75.30 | 69.60 | 62.00 | 51.60 | 37.80 | 26.80 | 17.50 | 11.00 | 3.00 | 0.70 | 0.00 | 0.00 | 0.00 |  |  |  |
|  | >10.0 | 10.70 | 16.40 | 24.40 | 33.70 | 45.70 | 61.20 | 72.60 | 82.20 | 88.90 | 97.00 | 99.30 | 100.00 | 100.00 | 100.00 |  |  |  |
| 5.0 | <5.0 | 6.50 | 5.90 | 3.50 | 2.10 | 1.70 | 0.50 | 0.30 | 0.10 | 0.10 | 0.00 | 0.00 | 0.00 | 0.00 | 0.00 | <0.001 |  |  |
|  | 5.0-10.0 | 72.80 | 65.80 | 56.80 | 49.10 | 38.40 | 25.60 | 17.60 | 10.40 | 6.90 | 1.60 | 0.50 | 0.00 | 0.00 | 0.00 |  |  |  |
|  | >10.0 | 20.70 | 28.30 | 39.70 | 48.80 | 59.90 | 73.90 | 82.10 | 89.50 | 93.00 | 98.40 | 99.50 | 100.00 | 100.00 | 100.00 |  |  |  |
| 5.5 | <5.0 | 4.70 | 4.00 | 0.10 | 1.10 | 0.80 | 0.30 | 0.30 | 0.10 | 0.10 | 0.00 | 0.00 | 0.00 | 0.00 | 0.00 | <0.001 |  |  |
|  | 5.0-10.0 | 62.50 | 53.60 | 4.50 | 37.70 | 29.00 | 17.00 | 10.70 | 6.50 | 4.30 | 0.90 | 0.40 | 0.00 | 0.00 | 0.00 |  |  |  |
|  | >10.0 | 32.80 | 42.40 | 5.30 | 61.20 | 70.20 | 82.70 | 89.00 | 93.40 | 95.60 | 99.10 | 99.60 | 100.00 | 100.00 | 100.00 |  |  |  |

^a^The frequency of administration is every 12 hour; C_VRC_, voriconazole concentration; PTA, probability of target attainment.

**Table S2. Tacrolimus PTA on day 3 based on different CREA and doses.**

| Dose^a^ | Level | CREA(μmol/L) | | | | | | | | | | | |
| --- | --- | --- | --- | --- | --- | --- | --- | --- | --- | --- | --- | --- | --- |
| (mg/q12h） |  | 40 | 100 | 160 | 400 | 600 | 800 | 1000 | 1600 | 1800 | 2000 | P |  |
|  |  | PTA(%) | | | | | | | | | | | |
| 0.5 | <5.0 | 100.00 | 100.00 | 100.00 | 100.00 | 100.00 | 100.00 | 100.00 | 100.00 | 100.00 | 100.00 | NA |  |
|  | 5.0-10.0 | 0.00 | 0.00 | 0.00 | 0.00 | 0.00 | 0.00 | 0.00 | 0.00 | 0.00 | 0.00 |  |  |
|  | >10.0 | 0.00 | 0.00 | 0.00 | 0.00 | 0.00 | 0.00 | 0.00 | 0.00 | 0.00 | 0.00 |  |  |
| 1.0 | <5.0 | 100.00 | 100.00 | 100.00 | 100.00 | 100.00 | 100.00 | 100.00 | 100.00 | 100.00 | 100.00 | NA |  |
|  | 5.0-10.0 | 0.00 | 0.00 | 0.00 | 0.00 | 0.00 | 0.00 | 0.00 | 0.00 | 0.00 | 0.00 |  |  |
|  | >10.0 | 0.00 | 0.00 | 0.00 | 0.00 | 0.00 | 0.00 | 0.00 | 0.00 | 0.00 | 0.00 |  |  |
| 1.5 | <5.0 | 100.00 | 100.00 | 100.00 | 99.70 | 99.90 | 99.80 | 98.80 | 97.50 | 97.10 | 96.50 | <0.001 |  |
|  | 5.0-10.0 | 0.00 | 0.00 | 0.00 | 0.30 | 0.10 | 0.20 | 1.20 | 2.50 | 2.90 | 3.50 |  |  |
|  | >10.0 | 0.00 | 0.00 | 0.00 | 0.00 | 0.00 | 0.00 | 0.00 | 0.00 | 0.00 | 0.00 |  |  |
| 2.0 | <5.0 | 100.00 | 100.00 | 100.00 | 94.60 | 90.50 | 83.90 | 81.40 | 73.00 | 69.40 | 73.50 | <0.001 |  |
|  | 5.0-10.0 | 0.00 | 0.00 | 0.00 | 5.40 | 9.50 | 16.10 | 18.60 | 26.90 | 30.40 | 26.30 |  |  |
|  | >10.0 | 0.00 | 0.00 | 0.00 | 0.00 | 0.00 | 0.00 | 0.00 | 0.10 | 0.20 | 0.20 |  |  |
| 2.5 | <5.0 | 100.00 | 99.20 | 94.40 | 68.50 | 58.60 | 50.80 | 49.30 | 41.80 | 39.20 | 40.00 | <0.001 |  |
|  | 5.0-10.0 | 0.00 | 0.80 | 5.60 | 31.20 | 41.30 | 49.00 | 49.50 | 55.70 | 57.90 | 56.50 |  |  |
|  | >10.0 | 0.00 | 0.00 | 0.00 | 0.30 | 0.10 | 0.20 | 1.20 | 2.50 | 2.90 | 3.50 |  |  |
| 3.0 | <5.0 | 99.60 | 89.60 | 71.60 | 37.10 | 28.80 | 24.40 | 23.90 | 20.80 | 17.50 | 19.60 | <0.001 |  |
|  | 5.0-10.0 | 0.40 | 10.40 | 28.40 | 62.00 | 69.00 | 70.60 | 69.30 | 67.80 | 69.40 | 68.50 |  |  |
|  | >10.0 | 0.00 | 0.00 | 0.00 | 0.90 | 2.20 | 5.00 | 6.80 | 11.40 | 13.10 | 11.90 |  |  |
| 3.5 | <5.0 | 96.30 | 63.60 | 45.40 | 14.40 | 12.80 | 11.90 | 10.70 | 9.60 | 8.50 | 9.60 | <0.001 |  |
|  | 5.0-10.0 | 3.70 | 36.40 | 54.60 | 80.20 | 77.70 | 72.00 | 70.70 | 63.40 | 60.90 | 63.90 |  |  |
|  | >10.0 | 0.00 | 0.00 | 0.00 | 5.40 | 9.50 | 16.10 | 18.60 | 27.00 | 30.60 | 26.50 |  |  |
| 4.0 | <5.0 | 85.70 | 36.10 | 21.80 | 7.40 | 5.80 | 4.70 | 5.00 | 4.40 | 3.90 | 4.20 | <0.001 |  |
|  | 5.0-10.0 | 14.30 | 63.80 | 76.50 | 77.40 | 69.40 | 62.90 | 60.30 | 53.20 | 48.20 | 50.50 |  |  |
|  | >10.0 | 0.00 | 0.10 | 1.70 | 15.20 | 24.80 | 32.40 | 34.70 | 42.40 | 47.90 | 45.30 |  |  |
| 4.5 | <5.0 | 65.40 | 18.20 | 9.10 | 3.80 | 1.50 | 2.20 | 2.80 | 2.30 | 1.90 | 2.40 | <0.001 |  |
|  | 5.0-10.0 | 34.60 | 81.00 | 85.30 | 64.70 | 57.10 | 48.60 | 46.50 | 39.50 | 37.30 | 37.60 |  |  |
|  | >10.0 | 0.00 | 0.80 | 5.60 | 31.50 | 41.40 | 49.20 | 50.70 | 58.20 | 60.80 | 60.00 |  |  |
| 5.0 | <5.0 | 41.30 | 9.10 | 3.80 | 1.30 | 0.60 | 1.20 | 1.10 | 1.20 | 0.80 | 1.20 | <0.001 |  |
|  | 5.0-10.0 | 58.60 | 86.90 | 81.30 | 49.60 | 41.40 | 33.60 | 32.90 | 28.00 | 27.30 | 26.40 |  |  |
|  | >10.0 | 0.10 | 4.00 | 14.90 | 49.10 | 58.00 | 65.20 | 66.00 | 70.80 | 71.90 | 72.40 |  |  |
| 5.5 | <5.0 | 23.40 | 4.50 | 1.40 | 0.70 | 0.20 | 0.30 | 0.40 | 0.80 | 0.50 | 0.80 | <0.001 |  |
|  | 5.0-10.0 | 76.20 | 85.10 | 70.20 | 36.40 | 28.60 | 24.10 | 23.50 | 20.00 | 17.00 | 18.80 |  |  |
|  | >10.0 | 0.40 | 10.40 | 28.40 | 62.90 | 71.20 | 75.60 | 76.10 | 79.20 | 82.50 | 80.40 |  |  |

^a^The frequency of administration is every 12 hour; CREA, serum creatinine; PTA, probability of target attainment. ^a^The frequency of administration is every 12 hour; C_VRC_, voriconazole concentration; PTA, probability of target attainment.

**Table S3.** Recommended Dosage Summary

| CVRC (μg/mL) Range | CREA (μmol/L) Range | PTA ≥ 70% Dosage Range | Recommended Dosage (mg/q12h) |
| --- | --- | --- | --- |
| 0 | 40-237 | 4.0-5.5 mg | 4.0-5.5 |
| 0 | 238-400 | 3.5-5.5 mg | 3.5-5.5 |
| 0 | 401-600 | 3.0-4.0 mg | 3.0-4.0 |
| 0 | 601-1000 | 2.0-3.5 mg | 2.0-3.5 |
| 0 | 1600-2000 | 1.5-2.0 mg | 1.5-2.0 |
| 0.5-1.0 | 237 | 2.5-4.5 mg | 2.5-4.5 |
| 1.5-2.0 | 237 | 2.0-4.5 mg | 2.0-4.5 |
| 2.5-3.0 | 237 | 1.5-3.5 mg | 1.5-3.5 |
| 3.5-4.5 | 237 | 1.5-2.5 mg | 1.5-2.5 |
| 5.0-7.0 | 237 | 1.0-1.5 mg | 1.0-1.5 |

Note: This table presents the recommended dosage range based on the interval forms of Voriconazole Concentration (CVRC) and Creatinine (CREA) levels, ensuring that PTA ≥ 70% is maintained.
